# Optimizing heat transfer performance in two-phase closed thermosyphons: A novel design and experimental evaluation

**DOI:** 10.1016/j.heliyon.2025.e42109

**Published:** 2025-01-19

**Authors:** Mohammad Khalili, Seyed Alireza Mostafavi, Seyed Mohammad Mousavi, Hossein Moghadamrad

**Affiliations:** a Department of Mechanical Engineering, Faculty of Engineering, Arak University, P.O.B. 38156-8-8349, Arak, Iran; b Institute of Advanced Technology, Arak University, P.O.B. 38156-8-8349, Arak, Iran

**Keywords:** Two-phase closed thermosyphon (TPCT), Thermal performance, Thermal resistance, Vapor-liquid interaction, Working fluids

## Abstract

Effective heat dissipation is crucial in various thermal management applications, including electronics, renewable energy systems, and heating and cooling systems. Two-phase closed thermosyphons (TPCTs) are recognized for their efficient heat transfer and have been widely adopted in these fields. This study presents a novel design for a TPCT that incorporates a unique internal cone-shaped tube at the evaporator. This innovative feature aims to minimize vapor-liquid interaction within the main tube, potentially leading to enhanced heat transfer efficiency. The proposed TPCT is evaluated against a conventional design using water and ethanol as working fluids. Thermal performance is assessed under varying heat inputs (50 W–250 W) and filling ratios (40 %, 55 %, 70 %, 85 %). The results indicate that the optimal filling ratio depends on the working fluid. Water exhibits the best performance at 55 % and 85 % filling ratios, whereas ethanol achieves its optimum at 70 %. Notably, at a 70 % filling ratio with a 50 W heat input, the novel TPCT design exhibits a significant 45.7 % reduction in thermal resistance compared to the conventional design. As expected, increasing heat input reduces thermal resistance but also elevates operating temperatures for both TPCTs. Notably, the novel TPCT demonstrates a significant improvement in thermal performance compared to the conventional design, particularly when using ethanol as the working fluid.

## Highlights

• Novel two-phase closed thermosyphon (TPCT) design: internal path liquid-vapor.
• Enhanced Heat Transfer Performance with Ethanol in Novel TPCT.
• Higher Heat Input Increases Temperature and Reduces Thermal Resistance.
• Novel TPCT Outperforms Conventional Design.

## 1 Introduction

The growing demand for sustainable energy solutions necessitates advancements in heat transfer technologies. Two-phase closed thermosyphons (TPCTs) have emerged as promising passive devices for heat transfer due to their reliance on natural convection and evaporation. TPCTs utilize a working fluid that evaporates in a heat source, absorbing heat. The vapor then rises to the condenser, where it condenses and releases the heat. Gravity facilitates the return of the condensate to the heat source, completing the cycle.

Extensive research has explored the thermal performance of TPCTs. Studies by Shiraishi et al. [1] investigated the influence of working fluids (water, ethanol, Freon 113) on thermal resistance, developing a successful mathematical model for TPCT heating performance prediction. Ma et al. [2] conducted both experimental and numerical analyses to evaluate TPCT thermal efficiency, immersing the evaporator in a hot water pool and cooling the condenser with circulating water. Their results showed that both total heat transfer and the overall heat transfer coefficient increased with higher hot water pool temperatures and decreased with lower cooling water temperatures. Han and Wang [3] identified that a 260 mm evaporator length at a 60-degree angle provided optimal heating performance. Similar findings by Goldoust et al. [4] supported this angle as achieving the highest thermal efficiency. In a different application, Yao et al. [5] implemented a cooling thermosyphon within a photovoltaic system, where the thermosyphon generated a buoyancy force of 8.22 N, resulting in a cooling water circulation speed of 0.011 m/s. Qin et al. [6] studied TPCTs with varying heights and filling ratios, finding that reducing the height significantly impacted thermal behavior. The thermosyphon with a 50 mm height, medium filling ratio, and the lowest input heat power demonstrated optimal performance, achieving a thermal resistance of 0.36 K/W. Liu et al. [7] examined an integrated thermosyphon system using a water condenser and latent heat energy storage condenser, exploring the influence of refrigerant filling ratio and inlet conditions. Their findings showed that as the filling ratio increased from 52.1 % to 80.2 %, the TWCLC cooling capacity decreased, and the system subcooling temperature was lower than a standard water-cooled thermosyphon. Mostafavi et al. [8] integrated a thermosyphon into a vehicle exhaust heating system for food storage, with a 75 % filling ratio achieving optimal performance, effectively heating the chamber to 48 °C within 25 min. Wang et al. [9] compared thermosyphons with corrugated and smooth evaporators, demonstrating that corrugated evaporators exhibited superior heat transfer capabilities, achieving a minimum total thermal resistance of 0.0243 K/W, lower than that of smooth evaporators under optimal test conditions.

Heat pipes (HPs) offer a versatile thermal management solution. Advancements in heat transfer technologies have led to various techniques to improve their performance, including fins (rectangular or combined with copper foam) and the use of wicks in the condenser section [[10], [11], [12]]. Beyond traditional cooling applications like CPU cooling [13,14], heat pipes find use in solar energy storage [15]. Researchers have explored various HP designs for specific applications. Wanison et al. [16] demonstrated the effectiveness of a parallel HP system, where multiple HPs share the same evaporator and condenser for efficient operation even under extreme conditions. Studies on non-conventional designs have yielded promising results. Chen et al. [17] found a cesium HP with a 68-degree tilt angle maintained consistent and uniform temperatures, while Im et al. [18] investigated oscillating HPs, known for their efficient heat transfer, but reported that smaller tilt angles negatively impacted performance. Wu et al. [19] designed a novel HP tool holder to reduce cutting temperatures and minimize cutting fluid consumption in machining. Similarly, Zhao et al. [20] explored HPs with disc-shaped evaporators for potential applications. Zhou et al. [21] observed that thermal insulation enhanced the performance of two-phase loop HPs, particularly at specific filling ratios. Wang et al. [22] used computational fluid dynamics (CFD) to analyze heat transfer in a two-phase ring thermosyphon, revealing boiling and condensation processes within the device. Vieira et al. [23] investigated the limitations of smaller diameter thermosyphons, which may not exhibit the same performance improvements with increasing heat input as traditional designs.

Several studies have explored how working fluids and design choices influence the performance of TPCTs. Kim et al. [24] investigated the impact of working fluids on heat transfer. They found that water experienced drying at high heat fluxes, leading to lower boiling heat transfer coefficients compared to acetone and HFE7100. This highlights the importance of selecting the appropriate working fluid for specific applications. Zhang et al. [25] focused on condenser design and operational factors. They developed a model to predict critical heat flux and liquid film thickness within a TPCT, demonstrating the influence of evaporator length, steam temperature, and condenser dimensions. Their work emphasizes the limitations imposed by condenser length on critical heat flux. Anandan et al. [26] conducted a combined analytical and experimental study, revealing that the evaporator's heat transfer coefficient increased with input power, while the condenser's coefficient decreased. This finding provides valuable insights into the internal heat transfer dynamics within a TPCT. Arat et al. [27] investigated the thermal performance of a closed-loop TPCT, finding that a 90-degree orientation yielded the lowest thermal resistance. Priya and Sakthivadivel [28] further explored design optimization by introducing a novel TPCT with a heterogeneous geometry. Their results demonstrated significant reductions in thermal resistance compared to conventional designs, particularly at specific angles. These studies showcase the potential for innovative TPCT designs to improve performance. Khalili et al. [29] investigated the thermal performance of an innovative TPCT equipped with an external vapor-liquid separator, comparing it to a conventional TPCT. Their findings indicate that optimal performance for all fluids tested occurred consistently at a 70 % filling ratio. While the conventional TPCT showed superior performance with methyl acetate, the new TPCT exhibited higher thermal efficiency when charged with water or ethanol.

Existing literature extensively highlights the efficient heat transfer capabilities of TPCTs. However, the interplay of vapor and liquid phases within the device often impedes optimal performance. This study introduces a novel TPCT design incorporating a dedicated internal pathway to mitigate vapor-liquid interactions. To comprehensively evaluate the proposed design, the influence of key parameters, including heat input, fluid filling ratio, and working fluid type, on the thermal performance of both conventional and modified TPCTs is investigated.

## 2 Experimental setup

### 2.1 Experimental setup and procedure

**Fig. 1:**
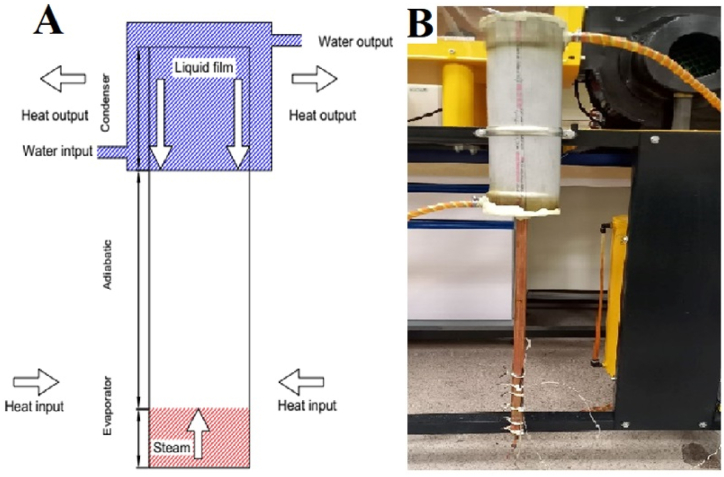
(A) Schematic, (B) Laboratory image of a conventional TPCT.

Fig. 1 illustrates the schematic and laboratory image of a conventional TPCT. The TPCT is fabricated from a 1000 mm long copper tube with an outer diameter of 22 mm and an inner diameter of 20 mm. It consists of three distinct sections: evaporator, adiabatic, and condenser, with respective lengths of 156 mm, 440 mm, and 404 mm. Copper caps are welded at both ends of the TPCT to enclose the working fluid. Additionally, a 6 mm diameter copper tube and a capillary tube are welded at both ends of the TPCT, serving the functions of evacuating air from the TPCT, introducing the working fluid, and draining the working fluid.

**Fig. 2:**
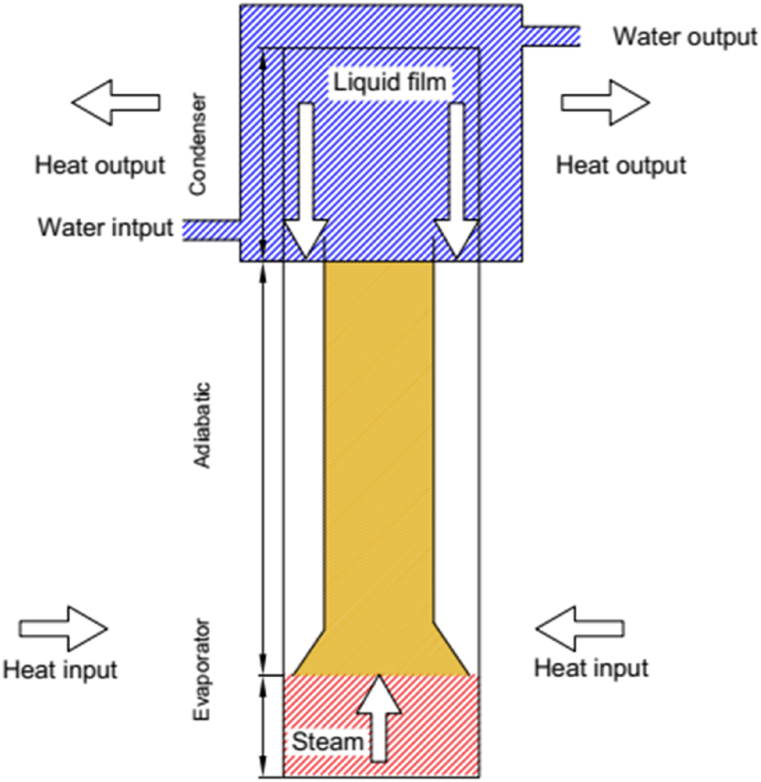
Schematic of a TPCT with an internal separation pathway.

**Fig. 3:**
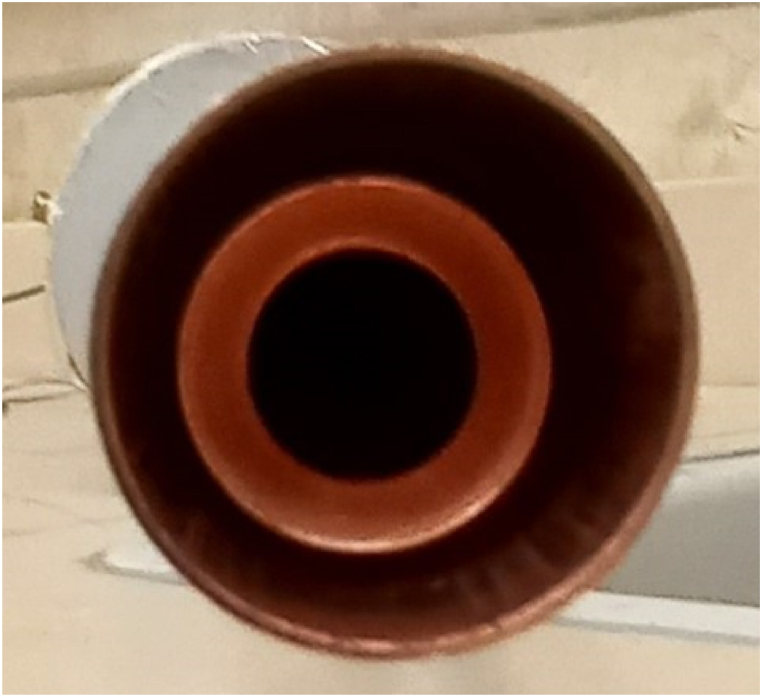
Internal separator path for vapor and liquid phases in the TPCT.

Fig. 2 illustrates the schematic of a newly designed TPCT incorporating an internal separator path. This TPCT maintains the core design principles of the conventional TPCT, with a key difference: the inclusion of a 540 mm long secondary tube with an outer diameter of 16 mm and an inner diameter of 15 mm. The initial section of this secondary tube is conically shaped and strategically positioned at the evaporator end within the main tube, as illustrated in Fig. 3. The key innovation of the proposed TPCT is the internal separator path. This path aims to minimize shear stress between the vapor phase rising from the evaporator and the liquid film returning from the condenser. In essence, the vapor travels from the evaporator to the condenser through the central separator pipe within the main pipe. The liquid film condenses on the main pipe's inner wall and returns to the evaporator along the periphery of the internal separator path, minimizing vapor-liquid interaction.

**Fig. 4:**
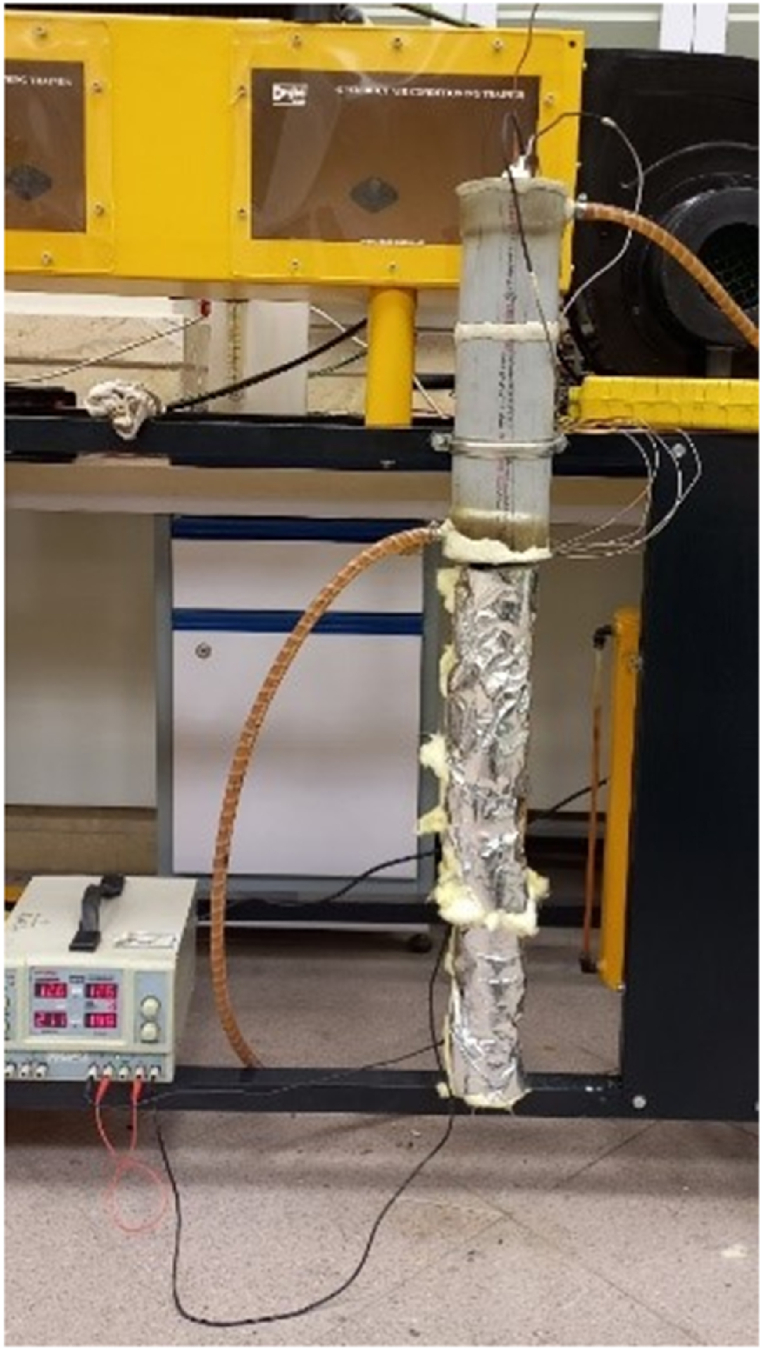
Image of a TPCT setup in the experiment.

Fig. 4 presents a schematic of the laboratory TPCT setup. The evaporator section is encircled by a heating element, with both ends connected to a DC power supply. This configuration allows for direct conversion of electrical current into heat via the element's resistance, effectively transferring the generated heat to the evaporator. To minimize heat loss, the element is insulated with glass wool. The condenser section is housed within a 405 mm long and 100 mm diameter PVC pipe. Water cooling is achieved by circulating water from a tank through the PVC pipe using a 50 W, 2400 rpm pump. The water enters from the bottom and exits at the top, ensuring efficient condenser cooling. Elastomeric insulation and glass wool are employed to thermally insulate the adiabatic section.

**Fig. 5:**
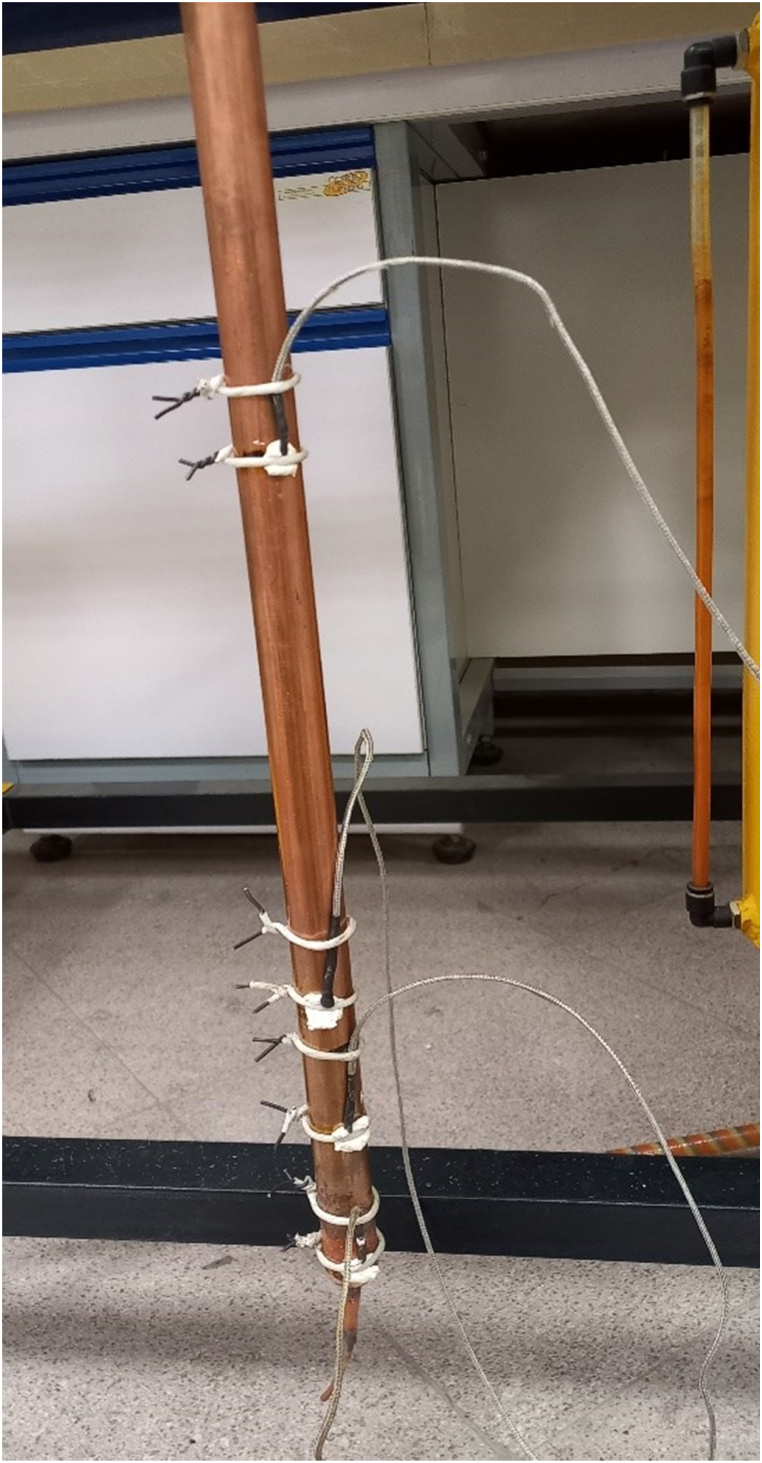
Placement of thermocouples on the TPCT tube.

As illustrated in Fig. 5, six K-type thermocouples monitor the external surface temperature of the TPCT tube. These are positioned at specific distances (10, 80, 146, 380, 750, and 850 mm) from the evaporator beginning. This configuration provides temperature data from three points within the evaporator, one in the adiabatic section, and two in the condenser section. Temperature readings are acquired every 5 s throughout the experiment and recorded using a 12-channel data logger. A VEN-115 model vacuum pump evacuates the TPCT. After evacuation, air and non-condensable gases are purged. Subsequently, the predetermined working fluid is charged into the TPCT at the desired filling ratio. Finally, the TPCT is hermetically sealed by welding to ensure a leak-proof system for testing.

The system's operational principle is succinctly outlined as follows: The experiment commences with activating the power supply to deliver heat input to the evaporator. Simultaneously, the water pump is engaged to facilitate heat rejection from the condenser. The applied heat input the copper tube and subsequently the working fluid within the evaporator. The working fluid temperature increases until it reaches the saturation temperature corresponding to the evaporator pressure, maximizing heat transfer before reaching this point. At the saturation temperature, the working fluid undergoes a phase change, absorbing latent heat of vaporization and transforming into vapor. This vapor, driven by higher evaporator pressure and buoyancy forces, migrates towards the condenser. After traversing the adiabatic section, the vapor enters the condenser. Here, the vapor cools down to the condenser's saturation temperature, mirroring the process in the evaporator. Upon reaching this temperature, the vapor releases the latent heat of condensation, forming a liquid film on the condenser's inner wall. Gravity then aids the return of this condensate film to the evaporator, completing the cycle for continuous operation.

The experiments evaluated various heat inputs ranging from 50 W to 250 W, filling ratios of 40 %, 55 %, 70 %, and 85 %, and employed two working fluids: water and ethanol.

### 2.2 Mathematical models

The governing equations for transient, compressible, and laminar vapor flow within the TPCT are expressed in cylindrical coordinates as follows [30]. The continuity equation is expressed as:

(1) $$\frac{\partial \rho_{v}}{\partial t}+\frac{1}{r}\frac{\partial }{\partial r}\left(r\rho_{v}v_{v}\right)+\frac{\partial \left(\rho_{v}w_{v}\right)}{\partial z}=0$$

Where $\rho_{v}$, $v_{v}$, and $w_{v}$ respectively represent vapor density, vapor axial velocity, and vapor kinematic viscosity. The momentum equations can be expressed as follows [30]:

(2) $$\frac{\partial v_{v}}{\partial t}+v_{v}\frac{\partial v_{v}}{\partial r}+w_{v}\frac{\partial v_{v}}{\partial z}=-\frac{1}{\rho_{v}}\frac{\partial P_{v}}{\partial r}+v_{v}\left[\frac{4}{3}\frac{1}{r}\frac{\partial }{\partial r}\left(r\frac{\partial v_{v}}{\partial r}\right)-\frac{4}{3}\frac{v_{v}}{r^{2}}+\frac{\partial_{v_{v}}^{2}}{{\partial z}^{2}}+\frac{1}{3}\frac{\partial_{w_{v}}^{2}}{\partial r\partial z}\right]$$

(3) $$\frac{\partial w_{v}}{\partial t}+v_{v}\frac{\partial w_{v}}{\partial r}+w_{v}\frac{\partial w_{v}}{\partial z}=-\frac{1}{\rho_{v}}\frac{\partial P_{v}}{\partial z}+v_{v}\left[\frac{1}{r}\frac{\partial }{\partial r}\left(r\frac{\partial w_{v}}{\partial r}+\frac{r}{3}\frac{\partial v_{v}}{\partial z}\right)+\frac{4}{3}\frac{\partial_{w_{v}}^{2}}{{\partial z}^{2}}+g\right]$$

The equation for vapor energy is given by

(4) $${\left(\rho c_{p}\right)}_{v}\frac{DT_{v}}{Dt}=\frac{1}{r}\frac{\partial }{\partial r}\left(rk_{v}\frac{\partial T_{v}}{\partial r}\right)+\frac{\partial }{\partial z}\left(k_{v}\frac{\partial T_{v}}{\partial z}\right)+\frac{DP_{v}}{Dt}+\mu_{v}\varphi$$

Where $c_{p}$, $T_{v}$, $k_{v}$, $P_{v}$, and $\mu_{v}$ are the specific heat capacity, vapor temperature, vapor thermal conductivity, vapor pressure, and vapor dynamic viscosity, respectively. In addition, φ represents the coefficient of viscosity loss and is expressed as follows [30]:

(5) $$\varphi =2\left[{\left(\frac{\partial v_{v}}{\partial r}\right)}^{2}+{\left(\frac{v_{v}}{r}\right)}^{2}+{\left(\frac{\partial w_{v}}{\partial z}\right)}^{2}\right]+{\left(\frac{\partial v_{v}}{\partial z}+\frac{\partial w_{v}}{\partial r}\right)}^{2}-\frac{2}{3}{\left[\frac{1}{r}\frac{\partial }{\partial r}\left(rv_{v}\right)+\frac{\partial w_{v}}{\partial z}\right]}^{2}$$

The filling ratio (FR) of the working fluid significantly impacts TPCT performance. It can be calculated using the following equation [30]:

(6) $$FR=\frac{V_{f}}{V_{e}}=\frac{4V_{f}}{\pi D^{2}L_{e}}$$

Where $V_{f}$ denotes the volume of the charged working fluid, $V_{e\;}$ signifies the volume of the evaporator section, and $L_{e}$ represents the length of the evaporator. Furthermore, the thermal resistance (R) is a crucial parameter for evaluating heat transfer efficiency within the TPCT. It can be determined using the following equation [30]:

(7) $$R=\frac{{\bar{T}}_{e}-{\bar{T}}_{c}}{Q}$$

Here, ${\bar{T}}_{e}$ represents the average evaporator temperature, ${\bar{T}}_{c}$ denotes the average condenser temperature, and Q signifies the heat input power to the evaporator [30].

## 3 Results and discussion

The thermophysical properties of working fluids significantly influence the performance of two-phase heat transfer devices like thermosyphons. Water and ethanol, with their distinct characteristics, can enhance efficiency under specific conditions and varying heat inputs.

**Fig. 6:**
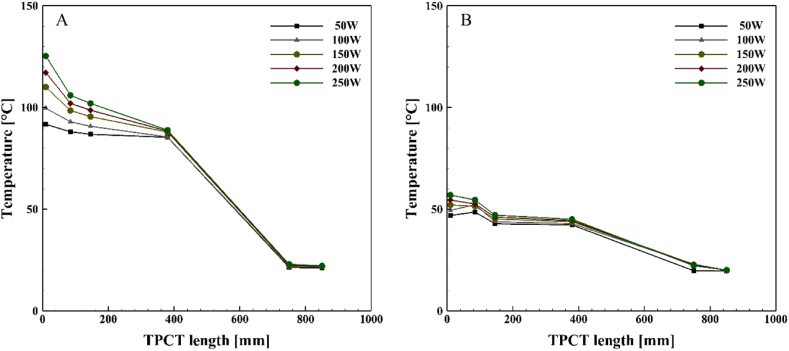
Temperature changes along the TPCT filled with water at different heat inputs for a filling ratio of 40 %: (A) Conventional TPCT, (B) Novel TPCT.

**Fig. 7:**
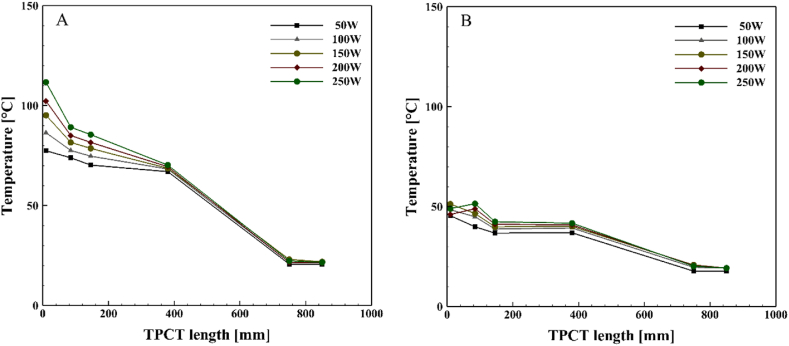
Temperature changes along the TPCT filled with water at different heat inputs for a filling ratio of 55 %: (A) Conventional TPCT, (B) Novel TPCT.

**Fig. 8:**
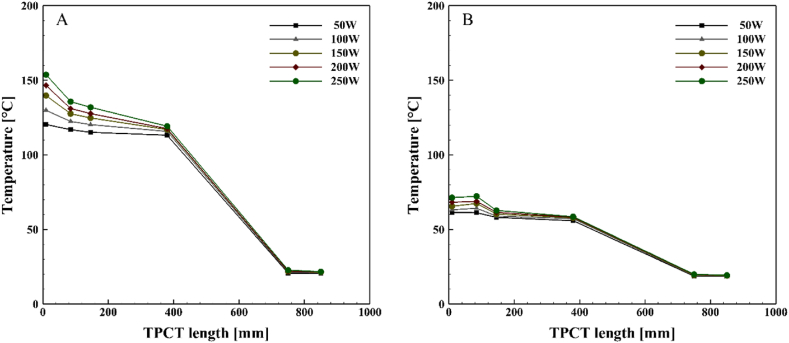
Temperature changes along the TPCT filled with water at different heat inputs for a filling ratio of 70 %: (A) Conventional TPCT, (B) Novel TPCT.

**Fig. 9:**
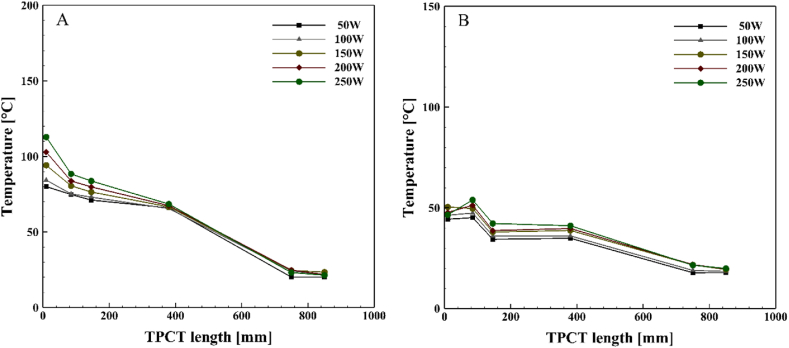
Temperature changes along the TPCT filled with water at different heat inputs for a filling ratio of 85 %: (A) Conventional TPCT, (B) Novel TPCT.

Fig. 6, Fig. 7, Fig. 8, Fig. 9 demonstrate the temperature distribution along both conventional and novel TPCTs filled with water at various filling ratios and distinct heat inputs. Observing the temperature distribution in the conventional TPCT across all tested filling ratios, it becomes evident that the temperature at the initial segment of the evaporator is higher compared to the middle and end temperatures. This temperature variation likely arises from insufficient liquid, causing the liquid layer on the evaporator wall to evaporate and induce dryness toward the end of the conventional TPCT. This pattern remains consistent across all tested heat inputs and filling ratios. The lowest temperature is observed in the condenser section, attributed to the continuous circulation of water. In the novel TPCT, the temperature at the initial segment of the evaporator has decreased, and the temperature at the middle segment and the initial segment of the evaporator is almost the same. However, the temperature at the end of the evaporator has decreased. This can be attributed to the presence of a cone-shaped structure that separates the vapor path in the evaporator, directing the vapor toward the center of the TPCT tube and reducing contact with the end of the evaporator. Generally, temperature changes in the conventional TPCT increased from 50 W to 250 W with rising heat input. In contrast, the novel TPCT exhibited varying temperatures at the evaporator's initial segment in some cases. Notably, temperature changes in the novel TPCT were significantly less compared to the conventional TPCT. The highest temperatures in both the conventional and novel TPCTs were associated with a filling ratio of 70 %. Specifically, at a filling ratio of 70 % and a heat input of 50 W, the temperature in the middle segment of the evaporator in the conventional TPCT increased by approximately 32.8 %, 58.1 %, and 56.2 % compared to filling ratios of 40 %, 55 %, and 85 %, respectively. Similarly, in the novel TPCT, under the same conditions, the temperature increased by 26.1 %, 53.6 %, and 35.9 %.

**Fig. 10:**
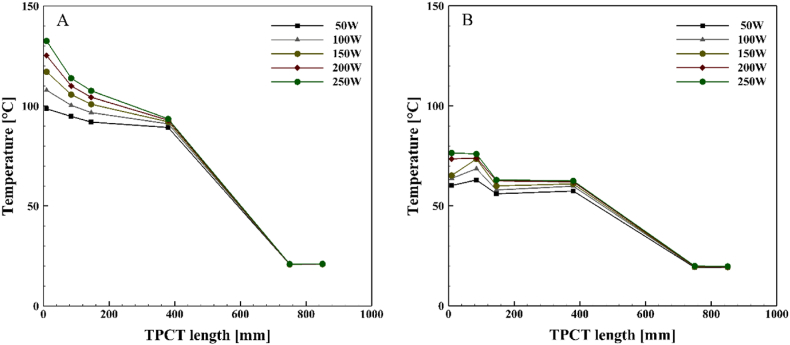
Temperature changes along the TPCT filled with ethanol at different heat inputs for a filling ratio of 40 %: (A) Conventional TPCT, (B) Novel TPCT.

**Fig. 11:**
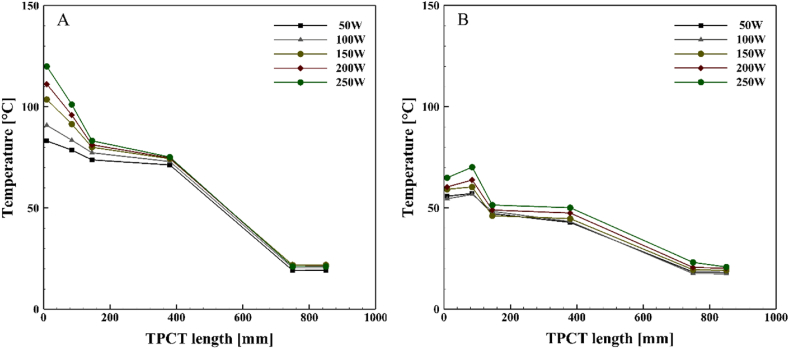
Temperature changes along the TPCT filled with ethanol at different heat inputs for a filling ratio of 55 %: (A) Conventional TPCT, (B) Novel TPCT.

**Fig. 12:**
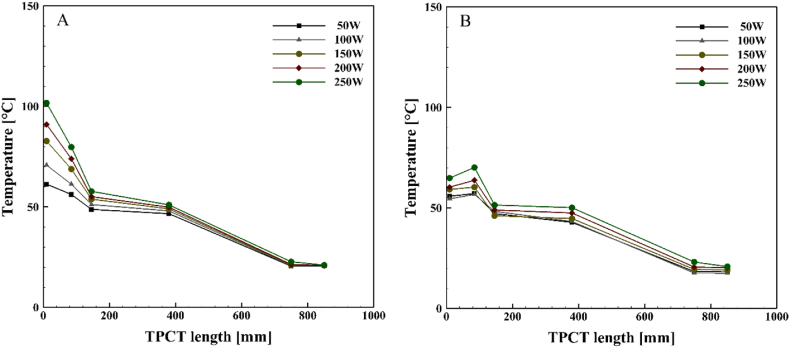
Temperature changes along the TPCT filled with ethanol at different heat inputs for a filling ratio of 70 %: (A) Conventional TPCT, (B) Novel TPCT.

**Fig. 13:**
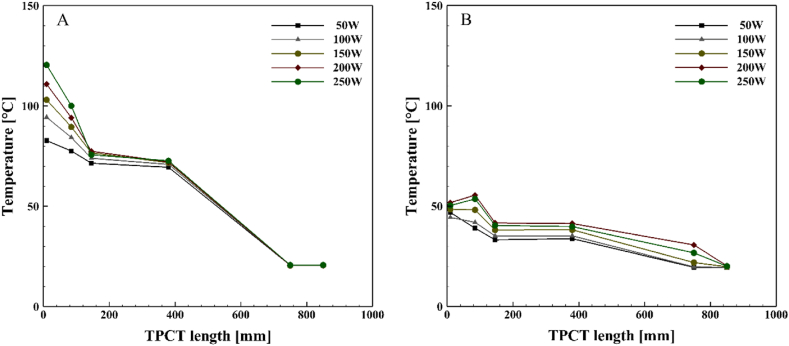
Temperature changes along the TPCT filled with ethanol at different heat inputs for a filling ratio of 85 %: (A) Conventional TPCT, (B) Novel TPCT.

Fig. 10, Fig. 11, Fig. 12, Fig. 13 illustrate the temperature distribution along both the conventional and novel TPCTs filled with ethanol at various filling ratios and distinct heat inputs. Similar to water, the temperature increases in both conventional and novel TPCTs filled with ethanol as the heat input increases from 50 W to 250 W. The highest temperatures in both the conventional and novel TPCTs were associated with a filling ratio of 40 %. Specifically, at a filling ratio of 40 % and a heat input of 50 W, the temperature in the middle segment of the evaporator in the conventional TPCT increased by approximately 20.6 %, 68.6 %, and 22.1 % compared to filling ratios of 55 %, 70 %, and 85 %, respectively. Similarly, in the novel TPCT, under the same conditions, the temperature increased by 10.3 %, 73 %, and 61.5 %. Due to the unique design of the novel TPCT, the movement of steam to the condenser through the central pipe is smoother and faster, leading to an increase in distillation rate and efficient reaching of the liquid film to the bottom of the evaporator. In contrast, the conventional TPCT does not reach the bottom of the evaporator due to a lower condensation rate of the liquid film, causing it to vaporize on the inner wall and return to the condenser. The temperature changes in the condenser section of the novel TPCT are more noticeable compared to the conventional TPCT. This is because the vapor and liquid phases are easily separated, resulting in more vapor reaching the condenser and raising its temperature. The significant temperature increase in the condenser section, especially at filling ratios of 70 % and 85 %, indicates a substantial separation of vapor and liquid phases. However, this temperature rise in the condenser section leads to an increased transfer of heat and energy from the evaporator to the condenser, causing a decrease in the temperature of the evaporator section in the novel TPCT compared to the conventional TPCT.

**Fig. 14:**
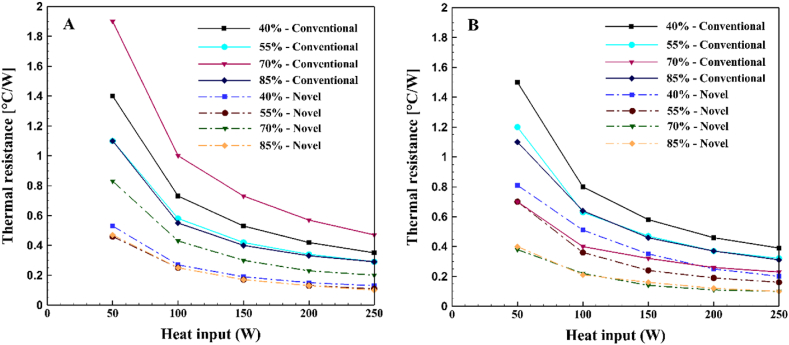
Comparison of thermal resistance in conventional and Novel TPCTs at different filling ratios and various heat Inputs for (A) water and (B) ethanol as working fluids.

The thermal resistance of a TPCT, which indicates the speed and efficiency of heat transfer from the evaporator to the condenser, is lower for more efficient heat transfer. Fig. 14 compares the thermal resistance of conventional and novel TPCTs filled with water and ethanol at different filling ratios and heat inputs. For water, the minimum thermal resistance in both TPCTs is associated with filling ratios of 85 % and 55 %. Conversely, the highest thermal resistance is linked to a filling ratio of 70 %. Specifically, the novel TPCT exhibited reductions of 36.3 %, 31 %, 23.8 %, 23.6 %, and 20.1 % in thermal resistance compared to the conventional TPCT, at a 55 % filling ratio and heat inputs of 50W, 100W, 150W, 200W, and 250W, respectively. When ethanol is used as the working fluid, the optimal filling ratio in both conventional and novel TPCT is 70 %. Notably, in the novel TPCT, a filling ratio of 85 % showed only a marginal difference in thermal resistance compared to the optimal filling ratio. The highest thermal resistance is observed at a filling ratio of 40 %, which can be attributed to the low level of filling in the TPCT. This is because at lower fill levels, the liquid inside the TPCT evaporates more quickly, leading to dry-out. This suboptimal performance at low filling ratios aligns with observations in Refs. [31,32]. In particular, when comparing the thermal resistance of the novel TPCT to the conventional TPCT at a 70 % filling ratio and heat inputs of 50W, 100W, 150W, 200W, and 250W, reductions of 45.7 %, 45 %, 56.3 %, 57.7 %, and 52 %, respectively, were observed.

The thermal resistance of the TPCT decreases as the heat input increases. This decrease is more pronounced in the conventional TPCT compared to the novel TPCT. The novel TPCT demonstrates the most optimal thermal performance. Additionally, when ethanol is used as the working fluid, the thermal resistance is lower compared to when water is used. However, as the heat input increases, the difference in thermal resistance between ethanol and water becomes smaller. Overall, the tests indicate that the novel TPCT outperforms the conventional TPCT in terms of thermal performance, especially when ethanol is employed as the working fluid.

## 4 Conclusion

The main objective of this research is to conduct an experimental analysis of the thermal performance of a novel designed two-phase closed thermosyphon (TPCT) with an internal vapor-liquid path and compare it with a conventional TPCT. In the proposed TPCT, a conical-headed tube is integrated within the main tube at the evaporator's end. In order to assess the performance of both TPCTs, several tests were conducted at filling ratios of 40 %, 55 %, 70 %, and 85 %, using heat inputs ranging from 50W to 250W, and two types of working fluids, water, and ethanol. Key findings from the tests are summarized as follows.

1. The optimal thermal performance for both TPCTs was observed at specific filling ratios: 55 % and 85 % for water working fluid, and 70 % for ethanol working fluid.
2. With an increase in heat input, the thermal resistance decreased and the temperature rose along both TPCTs.
3. The novel TPCT consistently exhibited superior thermal performance compared to the conventional design, particularly when using ethanol as the working fluid.

These findings contribute valuable insights into improving TPCT thermal performance, with potential applications across various industries that rely on efficient heat transfer systems. The novel TPCT's design, particularly the internal vapor-liquid separator, demonstrates promise for enhancing heat transfer efficiency, especially when using ethanol.

**Table:** Nomenclature

$C_{p}$	Specific heat capacity, J/kg⋅K	v	Axial velocity, m/s
FR	Filling ratio	w	Kinematic viscosity, m^2^/s
k	Thermal conductivity coefficient, W/m⋅K	μ	Dynamic viscosity, Pa⋅s
L	Length, m	ρ	Density, kg/m³
P	Pressure, Pa	**Subscripts**	
Q	Heat input, W	c	Condenser
R	Thermal resistance, °C/W	e	Evaporator
r	Radial coordinate, m	f	Fluid
T	Temperature, °C	v	Vapor
V	Volume, m^3^		

## CRediT authorship contribution statement

**Mohammad Khalili:** Visualization, Validation, Supervision, Software, Resources, Project administration, Methodology, Investigation, Funding acquisition, Formal analysis, Conceptualization. **Seyed Alireza Mostafavi:** Visualization, Supervision, Resources, Project administration, Methodology, Investigation, Funding acquisition, Formal analysis, Conceptualization. **Seyed Mohammad Mousavi:** Writing – original draft, Investigation, Data curation. **Hossein Moghadamrad:** Writing – review & editing, Writing – original draft.

## Declaration of competing interest

The authors declare that they have no known competing financial interests or personal relationships that could have appeared to influence the work reported in this paper.

>
